# ‘Inert’ co-formulants of a fungicide mediate acute effects on honey bee learning performance

**DOI:** 10.1038/s41598-023-46948-6

**Published:** 2023-11-09

**Authors:** Nicole S. DesJardins, Jessalynn Macias, Daniela Soto Soto, Jon F. Harrison, Brian H. Smith

**Affiliations:** School of Life Sciences, Arizona State University, Tempe, AZ USA

**Keywords:** Learning and memory, Environmental chemistry, Agroecology, Animal behaviour, Entomology

## Abstract

Managed honey bees have experienced high rates of colony loss recently, with pesticide exposure as a major cause. While pesticides can be lethal at high doses, lower doses can produce sublethal effects, which may substantially weaken colonies. Impaired learning performance is a behavioral sublethal effect, and is often present in bees exposed to insecticides. However, the effects of other pesticides (such as fungicides) on honey bee learning are understudied, as are the effects of pesticide formulations versus active ingredients. Here, we investigated the effects of acute exposure to the fungicide formulation Pristine (active ingredients: 25.2% boscalid, 12.8% pyraclostrobin) on honey bee olfactory learning performance in the proboscis extension reflex (PER) assay. We also exposed a subset of bees to only the active ingredients to test which formulation component(s) were driving the learning effects. We found that the formulation produced negative effects on memory, but this effect was not present in bees fed only boscalid and pyraclostrobin. This suggests that the trade secret “other ingredients” in the formulation mediated the learning effects, either through exerting their own toxic effects or by increasing the toxicities of the active ingredients. These results show that pesticide co-formulants should not be assumed inert and should instead be included when assessing pesticide risks.

## Introduction

Managed honey bee populations, in tandem with wild pollinators^1^, are experiencing high rates of loss both in the United States^2^ and globally^3^. The causal factors include habitat and forage loss, parasites and pathogens, climate change, and pesticides^1,4–7^. Pesticides are of particular concern, as managed honey bees have been exposed to increasing amounts of these chemicals over the past ~ 30 years^8^. Pesticide residues are frequently found inside hives at concerning levels^9–11^, and in some cases, greater residues in hives have correlated with colony mortality^12^.

At high enough doses, pesticides can cause lethality, but sublethal effects at lower doses can also substantially weaken colonies over time^13,14^. Sublethal effects can be physiological, demographic, or behavioral. Impaired learning performance is one example of a behavioral sublethal effect^15–17^. Learning is important because it is used in many aspects of bees’ daily lives; for example, when foraging for food in the outside world and navigating back to the colony^18–20^. Learning performance is commonly impaired in bees exposed to pesticides^16^, and it has been suggested that bees are especially vulnerable to pesticides because of their effects on learning, and by extension, foraging and navigation^21^.

The vast majority of studies focusing on pesticide effects on learning in honey bees have focused on insecticides^16^. The effects of other pesticides such as herbicides (although see^22^) and fungicides on honey bees are understudied in general^23,24^, and this is especially true in the learning literature. One study found a negative effect of acute exposure to the fungicide prochloraz on associative learning performance^25^. A study from our lab found a negative effect of chronic, colony-level exposure to the fungicide formulation Pristine on learning performance^26^, although a recent study found that chronic adult exposure to a similar formulation produced no effects^27^.

Here, we test the effects of acute exposure to the fungicide formulation Pristine (active ingredients: 25.2% boscalid, 12.8% pyraclostrobin) on olfactory associative learning performance in honey bees. Although we have already established that this fungicide impairs learning in individuals from chronically exposed colonies^26^, it remains unknown whether the formulation can also produce an acute effect. The presence of an acute, immediate effect on learning would strengthen the argument that Pristine is unsafe for honey bees by indicating that the fungicide could begin producing negative effects on individuals much more quickly. Additionally, it is unclear which component(s) of the formulation (boscalid, pyraclostrobin, or the co-formulants) are driving the effects on learning. The exact co-formulants of Pristine and most other agrochemicals are proprietary and generally not disclosed by manufacturers, so we test whether exposure to the active ingredients without co-formulants produces a learning deficit. Discovery of the offending agent(s) in the formulation will aid in the development of bee-safe alternatives.

## Methods

### Honey bee colonies and fungicide exposure

Returning foragers were collected from three managed honey bee colonies on the Arizona State University Tempe campus (33.41909, − 111.93140) at either 9 AM or 12 PM. Care was taken to ensure that each colony was sampled at roughly the same frequency. One colony was Carniolan (*Apis mellifera carnica*), and the other two were Italian (*Apis mellifera ligustica*). The experiment involving acute exposure to the Pristine formulation took place in February and March 2022, while the experiment involving acute exposure to boscalid and pyraclostrobin took place in March and April 2022. Conducting the two experiments in this sequence allowed us to first determine a dose of the formulation that impaired learning and then test the corresponding doses of the active ingredients only. Bees were brought into the lab, anesthetized on ice, and harnessed using plastic drinking straws and duct tape according to standard procedures for PER^17,26^ so that only their antennae and mouthparts were moveable.

Each bee was randomly assigned to a treatment group using a random number generator (random.org). For the first experiment, there were three treatment groups: untreated control (n = 104 individuals), 51.56 µg Pristine (n = 43 individuals), and 103.1 µg Pristine (n = 65 individuals). These doses were chosen to be in the sublethal range and represented approximately 1/16th (51.56 µg) and 1/8th (103.1 µg) of the LD_50_s for boscalid and pyraclostrobin, based on the United States EPA’s ECOTOX database^28^. It should be noted that this dose is substantially higher than what would be considered field-relevant, as Pristine occurs at concentrations between 3 and 24 ppm in bee-collected pollen^29^. A 24 ppm dose would be 0.00144 µg in 6 µL of the sugar solution. However, the field relevance of this scenario cannot be completely discounted, as bees may experience much higher doses if they forage on crops immediately after they have been sprayed. For the second experiment, there were two treatment groups: control (n = 70 individuals) and active ingredients (n = 77 individuals). Bees in the active ingredients group were given a mixture containing 26 µg boscalid and 13.2 µg pyraclostrobin, which corresponds to the doses of active ingredients in 103.1 µg Pristine. 1.0 M sucrose solutions were prepared, and then the proper amounts of the compounds were added (weighed out using an analytical balance) to create stock solutions. Bees were fed 6 µL of the appropriate solution immediately after being harnessed and assigned to a treatment group. They were then left in a humidified box for an hour before conditioning began.

### PER conditioning procedure

**Figure 1:**
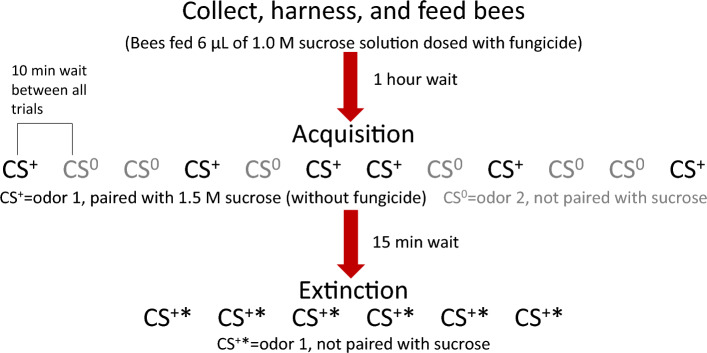
Phases of the experiment. Bees were captured, harnessed, and fed 6 µL of a sucrose solution containing the appropriate dose of the fungicide. After an hour-long rest, the acquisition phase began. This consisted of twelve trials (6 with the rewarded CS^+^ odor, 6 with the unrewarded CS^0^ odor). The extinction phase occurred after a 15-min rest. This consisted of 6 trials using the CS^+^ odor without reward.

The conditioning procedure was the same for both experiments (Pristine formulation and active ingredients). The equipment setup and basic protocol were the same as those described in previous publications^17,26^. As in DesJardins et al.^26^, we used a discrimination procedure with two different odors serving as the conditioned stimuli (CS). One served as the CS^+^ and was associated with a sugar reward, while the other served as the CS^0^ and was not associated with any reward or punishment. This procedure functioned as a built-in control and ensured that the bees learned to respond to the specific CS^+^ odor, as opposed to more general stimuli such as being moved into the arena^30^. There were six trials devoted to each odor (12 trials total), and they occurred in a pseudorandomized order (+ 0 0 + 0 +  + 0 + 0 0 +). See Fig. 1 for a summary of the experimental phases.

The chemicals 1-hexanol (Sigma-Aldrich, St. Louis, MO) and 2-octanone (Sigma-Aldrich, St. Louis, MO) were counterbalanced as the CS^+^ and CS^0^ odors. Odor cartridges were made by pipetting 7 µL of the appropriate undiluted chemical onto a strip of filter paper (Sigma-Aldrich, St. Louis, MO) and then placing that strip in a glass syringe (1 cc tuberculin syringe barrels, BD Medical, Franklin Lakes, NJ). During the conditioning procedure, odor cartridges were changed after every fifth trial.

The conditioning arena consisted of stands on which a harnessed bee and an odor cartridge could be mounted. The odor cartridge was attached to a programmable logic controller (Automation-Direct, Cumming, GA), which directed airflow through the cartridge at the appropriate times during the procedure. The arena was also hooked up to the laboratory exhaust system via dryer tubing. The vacuum was on for the entirety of the procedure to prevent odors from lingering in the arena.

The acquisition procedure was identical to the one described in DesJardins et al.^26^. At the beginning of each trial, a bee was placed inside the arena and allowed to acclimate for 25 s. For the next four seconds, air (flowing at 7 mL/s) was directed through the odor cartridge and toward the bee. During the last second of odor delivery, the bee was manually fed 0.4 μL of 1.5 M sucrose using a 0.2 mL Gilmont syringe (Cole-Parmer, Vernon Hills, IL). This occurred during CS^+^ trials only; during CS^0^ trials, the syringe was held near the bee’s head. A bee showing a conditioned response would extend its proboscis in response to the odor alone, before the sucrose solution was delivered. After this, the bee remained in the arena for another 30 s, and then the process was repeated with the next bee. We trained 10 bees at a time, and each trial took one minute, allowing for an inter-trial interval of 10 min for each bee.

At the conclusion of the acquisition phase, bees rested for 15 min before beginning the extinction phase. This phase consisted of six trials of the CS^+^ odor only, but the odor was not reinforced with the sugar solution. This allowed us to assess the bees’ initial memories of the association and whether extinction (learning to ignore a stimulus that no longer holds any biological value) occurred more quickly in any of the treatment groups.

### Statistical analyses

Data were analyzed in R version 4.3.0^31^. As in DesJardins et al.^26^, generalized linear mixed models (using a binomial distribution with a logistic link function) were created using the lme4 package^32^. Acquisition and extinction were analyzed in separate models, and the two experiments were also analyzed separately, leading to a total of four main models. Since the two experiments were conducted at slightly different times of year (February–March for the Pristine experiment and March–April for the boscalid/pyraclostrobin experiment), we created an additional two models to test for potential season-related differences in learning performance between the two control groups. In the main models, trial and treatment group were included as fixed effects, and individual was included as a random effect. For the acquisition models, in order to take into account the responses to both the CS^+^ and CS^0^ stimuli, the response variable was the discrimination index, or the difference between the response to the CS^+^ and the response to the CS^0^ in the corresponding trial. Thus, our statistics report a measure of the bees’ abilities to discriminate between the two odors during the acquisition phase. This calculation meant that the response value was either 1 (meaning the bee responded to the CS^+^ but not the CS^0^), 0 (meaning the bee responded to neither odor), or − 1 (meaning the bee responded to the CS^0^ but not the CS^+^). Since the generalized linear models we used only allowed for response variables ≥ 0, we changed the − 1 values to 0. This meant that each bee either correctly discriminated (value of 1) or did not (value of 0), which was the binary important to us in this scenario. In the seasonal models, experiment replaced treatment group as a fixed effect. Post-hoc pairwise comparisons, estimated marginal means with Tukey adjustment, were carried out using the emmeans package^33^. Raw data sets and code can be found in Supplementary Information 1.

## Results

### Acute exposure to the Pristine formulation

**Figure 2:**
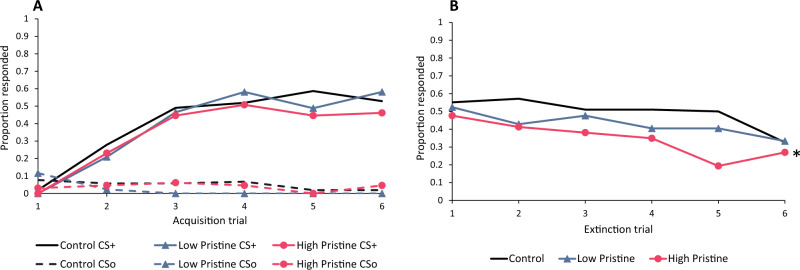
(**a**) Proportion of bees given an acute dose of the fungicide Pristine showing a learned response in a PER olfactory associative learning assay across six rewarded (solid lines) trials and six unrewarded (dashed lines) trials. Here, neither fungicide treatment group (low = 51.56 µg, high = 103.1 µg) performed differently than the controls. (**b**) Proportion of bees showing a learned response across six extinction trials. An asterisk (*) is placed next to the line denoting the high Pristine treatment group, which performed significantly worse than the control group across six trials.

There was no significant effect of Pristine exposure on discrimination between the two stimuli during the acquisition phase (χ^2^ = 1.30, *p* = 0.522), meaning that bees from both fungicide treatment groups learned as well as the controls (Fig. 2a). However, Pristine treatment produced a significant effect on performance during the extinction phase (χ^2^ = 6.94, *p* = 0.0312). Post-hoc pairwise comparisons revealed that the bees given the lower dose of Pristine (51.56 µg) performed similarly to controls. However, the bees given the higher dose (103.1 µg) performed significantly worse than controls, indicating that their memory was impaired (Fig. 2b).

### Acute exposure to boscalid and pyraclostrobin

**Figure 3:**
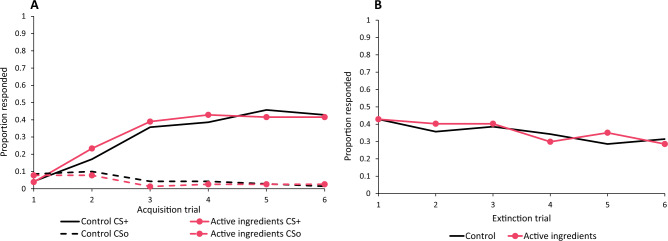
(**a**) Proportion of bees given an acute dose of the fungicides boscalid (26 µg) and pyraclostrobin (13.2 µg) showing a learned response in a PER olfactory associative learning assay across six rewarded (solid lines) trials and six unrewarded (dashed lines) trials. The fungicide group performed as well as the control group. (**b**) Proportion of bees showing a learned response across six extinction trials. Bees in the fungicide group performed similarly to the controls.

Bees exposed to a mixture of boscalid and pyraclostrobin (with doses corresponding to the higher dose of the formulation given in the previous experiment) performed similarly to controls in both the acquisition (χ^2^ = 1.05, *p* = 0.306, Fig. 3a) and extinction (χ^2^ = 0.174, *p* = 0.676, Fig. 3b) phases. This indicates that bees exposed to only the active ingredients had no deficits in associative learning or memory performance, unlike the bees exposed to the Pristine formulation.

Additionally, the seasonal models indicated a significant difference between the control groups for each experiment in both the acquisition (χ^2^ = 6.37, *p* = 0.0116) and extinction (χ^2^ = 7.06, *p* = 0.00789) phases. This indicates that the control group in the second experiment (boscalid/pyraclostrobin exposure, March–April) performed worse than the control group in the first experiment (Pristine exposure, February–March).

## Discussion

We exposed honey bees to acute sublethal doses of the fungicide formulation Pristine and then measured their performance in an olfactory associative learning task. Acquisition, or a bee’s ability to learn new information, was unaffected by Pristine regardless of dose. However, during the extinction phase (which happened 15 min after the conclusion of the acquisition phase), bees fed 103.1 µg Pristine performed more poorly than controls, indicating impaired memory. To determine which ingredients of the formulation were driving this effect, we also fed bees a solution containing the active ingredients (boscalid and pyraclostrobin) only, with doses corresponding to the amounts present in 103.1 µg Pristine. Treated bees performed as well as controls during both the acquisition and extinction phases. We conclude that acute exposure to a sublethal dose of Pristine can impair olfactory associative learning performance in honey bees, and that these effects might be mediated by the non-active “other ingredients”, which comprise 62 percent of the formulation^34^. This result suggests that fungicide co-formulants are not innocuous and that their potential toxicities should not be ignored, although they are often not considered by researchers and regulatory agencies^35^.

We acknowledge a weakness in our experimental design, in that the formulation was tested a few months before the active ingredients; an ideal experiment would have compared all treatment groups (Pristine, active ingredients, and controls) at the same time. We found a difference between control group performance in the two experiments, possibly due to seasonal differences in PER performance, as has previously been shown^36^. However, our main conclusion still stands—each fungicide-treated group was compared only to the controls trained during the same season.

The physiological mechanisms underlying the apparent acute effect of the inert ingredients in this fungicide formulation are unclear. In addition to boscalid and pyraclostrobin, the safety data sheet for Pristine^37^ lists the ingredients kaolin (< 5%), sodium-di-ethyl-hexyl-sulfosuccinate (0.1–1%), and ammonium sulfate (10–15%). These ingredients together do not account for the full 62% of “other ingredients”, so there are more (~ 41%) that are being kept as proprietary, which manufacturers are not required to disclose^38^. Sodium-di-ethyl-hexyl-sulfosuccinate is a surfactant, and a number of organosilicone and nonionic surfactants have been shown to impair PER learning performance in honey bees^39^. Kaolin is a hydrous aluminum silicate mineral, thought to be quite chemically inert^40^. However, kaolin has been used on crops as an ‘organic’ deterrent to insect herbivory, and it negatively impacts bumble bee survival^41^. Ammonium sulfate is often included as a co-formulant as it binds iron and calcium cations that can promote precipitation of active ingredients^42^; to our knowledge, there have been no studies of its toxicity to bees. There is an emerging picture that pesticide co-formulants can have negative effects on bees by themselves, including effects on development^43,44^ and mortality^45,46^. Another possibility is that the co-formulants/adjuvants are increasing the toxicity of the active ingredients in this fungicide, as many co-formulants and adjuvants facilitate active ingredient entry into biological tissues^47^. Supporting the possibility, in two studies with honey bees, adjuvants were non-toxic (no increased mortality), active ingredients increased mortality somewhat, and active ingredients together with adjuvants caused the greatest increases in mortality^48,49^. Also supporting the idea that the ‘inert’ ingredients may facilitate the toxicity of the active ingredients on learning, the neonicotinoid thiacloprid impaired learning and memory, but thiacloprid at the same does in its formulation Calypso more strongly impaired learning^50^. Moreover, the Calypso-treated bees had more thiacloprid in their tissues compared to bees given the active ingredient only, suggesting that the co-formulants may have facilitated thiacloprid uptake into bees^50^.

The results that we report here are slightly different from those of our previous study^26^, in which Pristine negatively affected acquisition as well as memory. The exposure scenario was quite different in the previous study, as we chronically (over a period of weeks) exposed entire colonies to field-relevant concentrations of the formulation (mixed into pollen patties). In comparison, the present study tested the effects of individual acute exposure to a much higher but still sublethal dose of Pristine (or its active ingredients), and found that memory, but not learning, was negatively impacted. The differing results suggest that the chronic effects of Pristine are at least partially developmentally mediated—the bees need to chronically consume the fungicide as adults and during development in order to experience the most severe effects^26^.

## Conclusion

We found that acute exposure to a sublethal dose of the fungicide formulation Pristine impaired memory, but this effect was not preserved when only the active ingredients boscalid and pyraclostrobin were tested. This suggests that the fungicide co-formulants, which are mostly trade secrets, are driving the effects on learning. These ingredients could be exerting toxic effects on their own, and/or they could be enhancing the toxicity of the active ingredients. This study adds to a growing body of literature^35,51–55^ suggesting that pesticide co-formulants and adjuvants are not inert and should never be discounted, although they often are by both researchers and regulatory bodies. Additionally, this study provides further evidence that the fungicide formulation Pristine is not safe for honey bees^26,29,56–62^; we have now shown that it can produce behavioral sublethal effects regardless of whether bees are exposed acutely or chronically.

### Supplementary Information

Supplementary Information 1.

Supplementary Information 2.

## Data Availability

All data generated or analyzed during this study are included in the Supplementary Information files of this article.
