# Supplementary material for: ‘Inert’ co-formulants of a fungicide mediate acute effects on honey bee learning performance
Source: Sci Rep. 2023 Nov 9;13:19458. doi: 10.1038/s41598-023-46948-6 (PMC10636155; doi:10.1038/s41598-023-46948-6)
Supplement: Supplementary file 2 — Supplementary Information 2. [file 41598_2023_46948_MOESM2_ESM.pdf]

Acute exposure R code

2023-10-13

```
#This code contains the analyses discussed in the article "‘Inert’ co-formulants of a fungicide mediate acute effects on honey bee learning performance" published in Scientific Reports

#Authors: Nicole S. DesJardins, Jessalynn Macias, Daniela Soto Soto, Jon F. Harrison, Brian H. Smith

#Please email Nicole DesJardins at ndesjard@asu.edu if you would like the R script associated with this markdown document.

###Packages and data
library(reshape2)
library(plyr)
library(lme4)
library(car)
library(emmeans)
formulationacq<-read.csv("Formulation acquisition.csv")
formulationext<-read.csv("Formulation extinction.csv")
ingredientsacq<-read.csv("Ingredients acquisition.csv")
ingredientsext<-read.csv("Ingredients extinction.csv")
controlacq<-read.csv("Control acquisition.csv")
controlext<-read.csv("Control extinction.csv")

###Formulation acquisition
##Formatting the data
formulationacq<-mutate(formulationacq, X1=X1.-X1o, X2=X2.-X2o, X3=X3.-X3o,
X4=X4.-X4o, X5=X5.-X5o, X6=X6.-X6o)
formulationacq<-subset(formulationacq, select=-c(X1., X2., X3., X4., X5.,
X6., X1o, X2o, X3o, X4o, X5o, X6o))
formulationacq<-melt(formulationacq, id.vars=c("ID", "group"))
colnames(formulationacq)[colnames(formulationacq)=="variable"]<-"trial"
colnames(formulationacq)[colnames(formulationacq)=="value"]<-"response"
formulationacq<-mutate(formulationacq, trial=as.numeric(trial))
formulationacq$response<-replace(formulationacq$response,
formulationacq$response<0, 0)

##GLMM
formulationacqglmm<-glmer(response~trial + group + (1|ID),
data=formulationacq, family=binomial(link=logit))
Anova(formulationacqglmm)

## Analysis of Deviance Table (Type II Wald chisquare tests)
##
## Response: response
```

```
##          Chisq Df Pr(>Chisq)
## trial 166.1035  1     <2e-16 ***
## group   1.3017  2     0.5216
## ---
## Signif. codes:  0 '***' 0.001 '**' 0.01 '*' 0.05 '.' 0.1 ' ' 1

###Formulation extinction
##Formatting the data
formulationext<-melt(formulationext, id.vars=c("ID", "group"))
colnames(formulationext)[colnames(formulationext)=="variable"]<-"trial"
colnames(formulationext)[colnames(formulationext)=="value"]<-"response"
formulationext<-mutate(formulationext, trial=as.numeric(trial))

##GLMM
formulationextglmm<-glmer(response~trial + group + (1|ID),
data=formulationext, family=binomial(link=logit))
Anova(formulationextglmm)

## Analysis of Deviance Table (Type II Wald chisquare tests)
##
## Response: response
##         Chisq Df Pr(>Chisq)
## trial 40.7718  1  1.711e-10 ***
## group  6.9351  2    0.03119 *
## ---
## Signif. codes:  0 '***' 0.001 '**' 0.01 '*' 0.05 '.' 0.1 ' ' 1

###Active ingredients acquisition
##Formatting the data
ingredientsacq<-mutate(ingredientsacq, X1=X1.-X1o, X2=X2.-X2o, X3=X3.-X3o,
X4=X4.-X4o, X5=X5.-X5o, X6=X6.-X6o)
ingredientsacq<-subset(ingredientsacq, select=-c(X1., X2., X3., X4., X5.,
X6., X1o, X2o, X3o, X4o, X5o, X6o))
ingredientsacq<-melt(ingredientsacq, id.vars=c("ID", "group"))
colnames(ingredientsacq)[colnames(ingredientsacq)=="variable"]<-"trial"
colnames(ingredientsacq)[colnames(ingredientsacq)=="value"]<-"response"
ingredientsacq<-mutate(ingredientsacq, trial=as.numeric(trial))
ingredientsacq$response<-replace(ingredientsacq$response,
ingredientsacq$response<0, 0)

##GLMM
ingredientsacqglmm<-glmer(response~trial + group + (1|ID),
data=ingredientsacq, family=binomial(link=logit))
Anova(ingredientsacqglmm)

## Analysis of Deviance Table (Type II Wald chisquare tests)
##
## Response: response
##         Chisq Df Pr(>Chisq)
## trial 84.2411  1     <2e-16 ***
## group  1.0475  1     0.3061
```

```
## ---
## Signif. codes:  0 '***' 0.001 '**' 0.01 '*' 0.05 '.' 0.1 ' ' 1

###Active ingredients extinction
##Formatting the data
ingredientsext<-melt(ingredientsext, id.vars=c("ID", "group"))
colnames(ingredientsext)[colnames(ingredientsext)=="variable"]<-"trial"
colnames(ingredientsext)[colnames(ingredientsext)=="value"]<-"response"
ingredientsext<-mutate(ingredientsext, trial=as.numeric(trial))

##GLMM
ingredientsextglmm<-glmer(response~trial + group + (1|ID),
data=ingredientsext, family=binomial(link=logit))
Anova(ingredientsextglmm)

## Analysis of Deviance Table (Type II Wald chisquare tests)
##
## Response: response
##         Chisq Df Pr(>Chisq)
## trial 13.2779  1  0.0002686 ***
## group  0.1741  1  0.6764958
## ---
## Signif. codes:  0 '***' 0.001 '**' 0.01 '*' 0.05 '.' 0.1 ' ' 1

###Control acquisition
##Formatting the data
controlacq<-mutate(controlacq, X1=X1.-X1o, X2=X2.-X2o, X3=X3.-X3o, X4=X4.-
X4o, X5=X5.-X5o, X6=X6.-X6o)
controlacq<-subset(controlacq, select=-c(X1., X2., X3., X4., X5., X6., X1o,
X2o, X3o, X4o, X5o, X6o))
controlacq<-melt(controlacq, id.vars=c("ID", "experiment"))
colnames(controlacq)[colnames(controlacq)=="variable"]<-"trial"
colnames(controlacq)[colnames(controlacq)=="value"]<-"response"
controlacq<-mutate(controlacq, trial=as.numeric(trial))
controlacq$response<-replace(controlacq$response, controlacq$response<0, 0)

##GLMM
controlacqglmm<-glmer(response~trial + experiment + (1|ID), data=controlacq,
family=binomial(link=logit))
Anova(controlacqglmm)

## Analysis of Deviance Table (Type II Wald chisquare tests)
##
## Response: response
##                Chisq Df Pr(>Chisq)
## trial      132.6833  1    < 2e-16 ***
## experiment   6.3653  1    0.01164 *
## ---
## Signif. codes:  0 '***' 0.001 '**' 0.01 '*' 0.05 '.' 0.1 ' ' 1
```

```r
###Control extinction
##Formatting the data
controlext<-melt(controlext, id.vars=c("ID", "experiment"))
colnames(controlext)[colnames(controlext)=="variable"]<-"trial"
colnames(controlext)[colnames(controlext)=="value"]<-"response"
controlext<-mutate(controlext, trial=as.numeric(trial))

##GLMM
controlextglmm<-glmer(response~trial + experiment + (1|ID), data=controlext,
family=binomial(link=logit))
Anova(controlextglmm)

## Analysis of Deviance Table (Type II Wald chisquare tests)
##
## Response: response
##              Chisq Df Pr(>Chisq)
## trial      23.0173  1  1.605e-06 ***
## experiment  7.0592  1   0.007886 **
## ---
## Signif. codes:  0 '***' 0.001 '**' 0.01 '*' 0.05 '.' 0.1 ' ' 1
```
